# Gene therapy: progress and predictions

**DOI:** 10.1098/rspb.2014.3003

**Published:** 2015-12-22

**Authors:** Mary Collins, Adrian Thrasher

**Affiliations:** 1 Division of Infection and Immunity, University College London, Gower Street, London WC1E 6BT, UK; 2 Institute of Child Health, University College London, Gower Street, London WC1E 6BT, UK; 3 Division of Advanced Therapies, National Institute for Biological Standards and Control, Blanche Lane, Potters Bar, Herts EN6 3QG, UK

**Keywords:** gene therapy, evolutionary medicine, personalized medicine

## Abstract

The first clinical gene delivery, which involved insertion of a marker gene into lymphocytes from cancer patients, was published 25 years ago. In this review, we describe progress since then in gene therapy. Patients with some inherited single-gene defects can now be treated with their own bone marrow stem cells that have been engineered with a viral vector carrying the missing gene. Patients with inherited retinopathies and haemophilia B can also be treated by local or systemic injection of viral vectors. There are also a number of promising gene therapy approaches for cancer and infectious disease. We predict that the next 25 years will see improvements in safety, efficacy and manufacture of gene delivery vectors and introduction of gene-editing technologies to the clinic. Gene delivery may also prove a cost-effective method for the delivery of biological medicines.

## 1. Introduction

Before the first human coding sequence had been determined, there was already speculation about the prospects for gene therapy. A prescient editorial published in *Science* in 1971 outlined many of the problems that would face clinical gene therapy, including construction of safe viral gene delivery vectors and efficient gene delivery to enough patient cells to correct the inherited gene defect [1]. Some 40 years later, the same issues persist but substantial progress has been made. This review will discuss current developments in delivery technology, describe clinical achievements to date and finish with speculation on future prospects.

In 2015, there is no lack of information on the structure of the human genome. The first draft human genome sequence was published in 2001, with an estimate of 30 000–40 000 protein-coding sequences [2]. Current estimates are closer to 20 000 protein-coding genes, with an expanding number of functional, non-coding RNAs. Identifying the molecular basis of inherited genetic disorders has become much easier; at the time of writing this has been achieved for 3674 human phenotypes, the majority being single-gene mutations [3]. Information on the remaining 1765 described phenotypes with Mendelian inheritance cannot be long in coming. Thus, there are potentially several thousand severe recessive genetic disorders, for which gene replacement therapy could be a treatment. Gene replacement therapy is a simple concept: insert a correct copy of the defective gene into the necessary cells. This review discusses current progress in some degree of detail because bringing this simple concept to fruition is technically demanding and has taken much longer than originally anticipated.

In contrast to the simple concept of gene replacement therapy, the majority of gene therapy clinical trials to date have involved ‘gene addition’. Over 60% of trials have been for cancer, probably because of the large numbers of affected patients, oncology's track record in innovative therapy and the seriousness of the disease [4]. Gene therapy may also provide an effective treatment for other acquired diseases; for example, it is one of a number of new ideas for Parkinson's disease (reviewed in [5]). In the case of infection, gene therapy approaches include immune cell engineering [6], antibody gene expression [7] and gene editing to remove pathogen receptors [8]. Gene therapy research has also contributed viral vectors being applied to vaccination for infectious diseases and cancer [9].

Gene replacement therapy has the relatively defined objective of sufficient gene expression in enough appropriate cells to ameliorate or correct the phenotype. By contrast, the options for gene addition therapy are essentially unlimited. Indeed, there are even concerns that gene addition may be used illicitly, for example, to express erythropoietin in the muscles of athletes [10].

## 2. Gene therapy remains a delivery challenge

**Figure 1.**
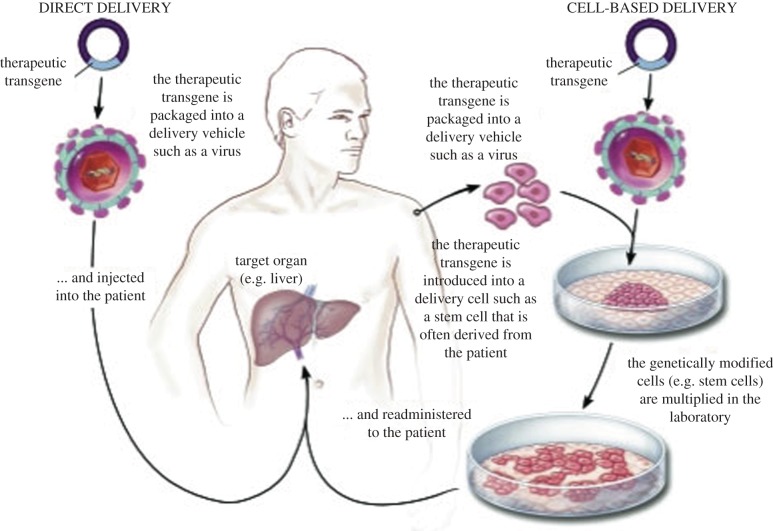
Direct and cell-based gene delivery (adapted from [89]). (Online version in colour.)

The most elegant method of gene delivery, in terms of defined composition and manufacturing reproducibility, would involve synthetic particles, for example, using lipids or polymers to carry DNA. However, these methods have not yet achieved efficient uptake and sustained gene expression *in vivo.* So the gene replacement therapy trials that have demonstrated clinical benefit, discussed in §§3–6, have all used viral vectors for gene delivery, because viruses are highly adapted for gene delivery to their host cells. These have either involved direct viral vector injection to target tissues such as liver, or modification of cells in culture by viral vectors, followed by cell expansion and injection (figure 1).

**Figure 2.**
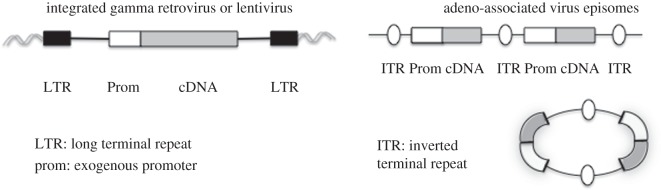
Viral vector genomes in target cells.

Viruses of the family *retroviridae* integrate their genome into host cell DNA as part of their life cycle [11] (figure 2). This means that the integrated provirus is transmitted to daughter cells when the infected cell divides. A murine leukaemia virus (MLV) was the first retroviral genome to be engineered to carry a foreign gene, herpes simplex virus thymidine kinase [12]. Deletion of the sequence required for packaging the viral RNA into particles allowed viral genes required for particle production to be provided *in cis* to replication-defective MLV vectors carrying no viral genes [13]. Viral packaging cell lines were then constructed with the viral genes expressed from two segments of DNA, essentially eliminating the risk of recombination with the vector to generate replication-competent virus [14,15]. The ability of MLV vectors to deliver genes to mouse and human bone marrow stem cells was very soon demonstrated [16,17]. This was because a number of inherited diseases could be cured by bone marrow transplantation if a suitable donor was available; therefore, gene therapy using the patient's own cells engineered to carry a correct copy of the faulty coding sequence seemed an attractive option for patients without a suitable donor. The feasibility of gene transfer to patients was first demonstrated by Rosenberg *et al.*, who used an MLV vector to introduce the neomycin-resistance gene into tumour-infiltrating lymphocytes before infusing the cells into five patients with advanced melanoma [18].

One drawback of MLV vectors is that they can only transduce dividing cells, because the MLV core needs nuclear envelope breakdown to access chromatin [19]. However, vectors engineered from HIV can transduce most non-dividing cells and tissues [20], as the pre-integration complex of nucleic acid and protein from HIV is imported across the nuclear membrane (reviewed in [21]). Unlike MLV vectors, HIV-based vectors can incorporate genomic sequences containing potential splice sites. For example, MLV vectors containing globin genomic sequences required as powerful tissue-specific enhancers proved unstable because splicing of the vector genome occurred [22]. However, the HIV genome contains multiple splice sites, so it encodes the Rev protein to facilitate expression of unspliced RNA [23]. In §3, the use of MLV- and HIV-based vectors for the treatment of inherited disorders by bone marrow transplantation will be discussed.

Vectors based on the non-pathogenic human parvovirus adeno-associated virus (AAV) have also been used for the treatment of inherited disorders, in this case by local or systemic direct vector injection, as discussed in §§4 and 5. AAV replicates only in the presence of a helper virus, in humans usually an adenovirus. There are a number of capsid varieties of AAV (serotypes) and most individuals have been exposed to at least one of these [24]. In the absence of helper virus, AAV integrates its genome into cellular DNA [25], remarkably into a preferential single locus (AAVS1) on human chromosome 19 [26]. Initial vectors replaced the AAV capsid gene with a transgene but left the AAV Rep coding sequence, which directs site-specific integration [27]. However, the coding capacity of AAV is relatively small so in general *cap* and *rep* genes are removed, leaving the AAV inverted terminal repeat sequences in the vector, with *cap, rep* and adenovirus helper functions supplied *in trans* [28,29]. These AAV vectors do not integrate efficiently so they are not maintained in dividing cells; however, they do modify non-dividing cells stably in tissues *in vivo* [30,31]. After injection into muscle the AAV vector genome forms double-stranded DNA episomal circles or concatamers [32] (figure 2). It has been shown that rare AAV integrations can cause hepatocellular carcinoma in mice, but the vector design can be modified to reduce this risk [33]. Completion of second-strand DNA synthesis for the single-stranded genome in the vector particles is a rate-limiting step for AAV vector gene expression [34]. Vectors that package double-stranded DNA have been developed to overcome this [35]. In §4, the clinical use of AAV vectors injected locally in the eye, or systemically to target the liver, is described. Interestingly, AAV has also been shown to mediate homologous recombination at surprisingly high frequencies [36].

Recently, several methods for editing the cellular genome have been described. For inherited disorders with a dominant defective gene, such as Huntington's disease, this offers the opportunity to disrupt expression of the pathogenic gene in the tissues where it causes the worst symptoms. There is also the possibility of repairing genes in recessive genetic disorders. In the first of these new technologies synthetic combinations of zinc finger DNA-binding domains, targeting a mutated human genomic sequence, were coupled to an endonuclease domain to generate a zinc finger nuclease (ZFN). When expressed in human cells, this enzyme induced a double-stranded break in the genomic target sequence, which was repaired by homologous recombination when a correct template was supplied [37]. Homing endonucleases, or meganucleases, cut genomic DNA within the cells that synthesize them at very low frequency. Repair by the host cell can result in copying the homing endonuclease gene into the cleavage site, hence ‘homing’. The I-SceI meganuclease has been engineered to target specific sites in the mammalian genome [38]. Transcription activator-like effectors (TALEs) are transcriptional activators from *Xanthomonas* plant pathogens, comprising a series of approximately 30 amino acid repeats, each of which binds to a single target base in a DNA sequence. Engineered TALE-nuclease chimaeras (TALENs) can also be used for site-specific genome cleavage and repair in mammalian cells [39]. The advent of these methods for editing the cellular genome has also allowed targeted integration of expression cassettes to ‘safe harbours' such as the AAVS1 site on chromosome 19 or the endogenous locus. To achieve this, the expression cassette must be flanked with DNA surrounding the nuclease cleavage site to direct homologous repair and also be delivered efficiently, for example with a non-integrating lentiviral vector [40].

Most recently, a bacterial clustered regularly interspaced short palindromic repeats (CRISPR) RNA together with a CRISPR-associated (Cas) protein has been used to target and mutate a mammalian cell locus [41]. As this latter technology uses an RNA sequence to specify the genomic locus, it is less cumbersome than engineering a protein with novel DNA binding and cleavage specificity. This technology has been used in cell culture to inactivate integrated HIV genomes [42], to make cells resistant to HIV by disrupting the CCR5 co-receptor [43], and to correct the cystic fibrosis transporter mutation in patient cells [44]. In adult mice, it has been used to repair a fumarylacetoacetate hydrolase mutation in mouse liver, because corrected cells can be selected *in vivo* [45]. An efficient delivery technology will be needed for clinical gene therapy using CRISPR/Cas, and the off-target toxicity of cleavage elsewhere in the genome will need critical evaluation.

## 3. Replacement gene therapy using bone marrow transplantation

Patients with adenosine deaminase (ADA) deficiency accumulate toxic purine metabolites. Their most immediate problem is a severe combined immune deficiency (SCID), resulting in multiple life-threatening infections in early childhood. Before the advent of gene therapy, effective treatment involved either bone marrow transplantation if a suitable donor was available, or regular injection of recombinant enzyme. The first successful gene therapy treated two children without suitable bone marrow donors, who did not have funding for recombinant enzyme therapy. Previous attempts had largely failed because insufficient numbers of cells were engrafted, but in this pioneering study, patients also received low-intensity myelosuppression. Bone marrow stem cells from the patients were isolated using magnetic beads coated with an antibody to the surface marker CD34. They were cultured for 4 days with cytokines and an MLV vector carrying an ADA cDNA, then reinfused. At the time of the first report, 1 year later, the patients had substantially reconstituted lymphoid cells and immune responses [46]. To date 40 patients have been treated with similar MLV vectors; the best results are similar to those of matched bone marrow transplantation, and better in terms of cost and immune reconstitution than enzyme replacement [47–49]. Future treatment of ADA-SCID is likely to use HIV-based lentiviral vectors because of greater efficiency in transduction of bone marrow stem cells and improved safety, and these studies are under way (see below).

A trial of gene therapy for a second inherited immune deficiency, SCID-X1, was reported at the same time as the first ADA-SCID trial [50]. In this X-linked disease, boys lack expression of the common gamma chain of a cytokine receptor, critical for T-cell development. The gene-corrected cells, expressing the receptor, are able to proliferate and differentiate competitively so no elimination of the patients' haematopoietic system was necessary. In the first report, 2 years after gene therapy, T cells had developed in the five boys and their immune systems functioned relatively normally. However, in this trial, and a similar study carried out at Great Ormond Street Hospital, a number of patients developed a T-cell leukaemia that was caused by the inserted MLV gene delivery vector switching on an adjacent oncogene [51,52]. So for SCID-X1 the majority of the 19 younger patients gained significant clinical benefit, but five developed leukaemia, of whom four were successfully treated and recovered immunity without the need for further intervention. Clearly, the long terminal repeat (LTR) of the MLV vector contains a powerful enhancer, which can cause insertional oncogenesis, for example, when the wild-type virus infects tumour-prone mice [53]. A more recent trial treating SCID-X1 has used an MLV vector without the enhancer in the LTR. Thus far the patients have reconstituted T cells with no leukaemia [54]. It is not totally clear why the LTR-containing MLV vector caused insertional oncogenesis in SCID-X1 but not ADA-SCID, but similar insertional oncogenesis in patients treated for chronic granulomatous disease and Wiskott–Aldrich syndrome suggests that ADA deficiency offers some intrinsic protection [55,56]. A new clinical trial using a lentiviral vector to treat Wiskott–Aldrich syndrome has not reported insertional oncogenesis [57].

Bone marrow can also be used to treat neurometabolic disorders, as migroglial cells or their precursors are able to cross the blood–brain barrier. For example, X-linked adrenoleukodystrophy (ALD) is caused by the lack of a transporter involved in the peroxisomal degradation of very long-chain fatty acids by oligodendrocytes and microglia. This disrupts maintenance of myelin by these cells, resulting in serious neurological consequences and death in childhood. Metachromatic leukodystrophy is a deficiency of arylsulfatase A that causes build-up of sulfatide leading to cytotoxicity in oligodendrocytes and microglia. ALD can be treated by bone marrow transplantation, while the most severe MLD cannot. In both conditions, gene therapy using lentiviral vectors to transduce bone marrow stem cells is effective; indeed the supraphysiological level of gene expression achieved with gene therapy in early-onset MLD makes it the only effective treatment [58,59].

The improved safety of lentiviral vectors compared with LTR-containing MLV vectors in bone marrow gene therapy is because the lentiviral vectors have been engineered to remove any enhancer activity from the LTR, reducing the risk of activation of expression of adjacent genes. However, when a lentiviral vector was used to treat a patient with β-thalassaemia a different mechanism of cellular gene upregulation was seen, involving truncation of a cellular mRNA by provision of a splice acceptor in the lentiviral vector (see above) [60]. Ongoing work in lentiviral vector design aims to eliminate splice donors and acceptors [61,62].

## 4. Direct injection of adeno-associated virus vectors for gene replacement therapy

The eye is an attractive target for direct gene delivery; it is accessible, one eye only can be treated in case of toxicity, only a small amount of vector is needed, and the eye is an immune-privileged site where inflammation and immunity are suppressed. Furthermore, the retina is a complex tissue; approximately 1 in 3000 of the population suffers from an inherited defect in one of over 60 genes that leads to retinal degeneration and blindness [63]. The first disease to be tackled by gene therapy was an *RPE65* gene defect. The RPE65 protein is expressed in the retinal pigment epithelium and is needed for conversion of all-trans retinal, generated during photoreceptor response to light, back to 11-cis retinal. Three groups have injected very similar AAV serotype 2 (AAV2) vectors sub-retinally and have reported results on a total of 21 patients [64–66]. The treatment was safe, and some improvements in retinal function and visual performance were found. Improvement of visual acuity has also been reported in patients treated for a deficiency in the Rab escort protein REP1 [67]. The safety of these trials will encourage treatment of younger patients, where more improvement is expected, and ongoing pre-clinical work is developing therapies for other retinopathies.

AAV vectors have also been used for treatment of patients with haemophilia B, a deficiency of Factor IX (F.IX) of the blood-clotting cascade. These patients are reliant on prophylactic or on-demand plasma, or recombinant F.IX injection, to prevent spontaneous bleeding, and still suffer progressive joint damage and life-threatening conditions such as intracranial haemorrhage. Because of the cost this therapy is not available in less-developed countries. As many cells can secrete F.IX when transduced by AAV, the first clinical trial used AAV2 encoding F.IX injected into muscle. Levels of F.IX about 1% of normal could be detected, but clinical benefit was limited [68]. The same investigators then infused a higher dose of the same vector into the hepatic artery, with the aim of transducing hepatocytes the cells that normally produce F.IX. In this case, therapeutic levels of F.IX were achieved at the highest vector dose; however, these declined over the next two months because of an immune response to the AAV2 capsid, which eliminated the transduced hepatocytes [69]. More recently, the use of a self-complementary AAV8 vector injected intravenously showed great promise, with a number of patients discontinuing prophylaxis for up to 3 years thus far [70,71]. The improved results with this vector are due to more efficient transduction with the self-complementary vector and the use of the AAV8 serotype, which is more efficient in gene delivery to hepatocytes. This allowed intravenous administration with a relatively moderate vector dose. Exposure to AAV8 in the population is also much lower than that to AAV2, and there was no evidence of prior immunity to AAV8 in the patients in the latest clinical trial. Some patients treated at the highest vector dose developed signs of liver inflammation, which could be treated with a course of steroids. These encouraging results open up the possibility of other gene therapy applications using the liver as a site of production and secretion.

## 5. Gene therapy to treat cancer and infectious disease

Perhaps because of the seriousness of the condition, cancer physicians have instigated by far the greatest number of gene therapy clinical trials to date. A number of these are cancer vaccine trials using engineered viral vectors. These are often classified as gene therapy, unlike the use of similar vectors as prophylactic or therapeutic vaccines for infectious disease. Correction of the tumour cell genotype, for example, by restoration of tumour suppressor gene function has been proposed as on approach for cancer gene therapy [72]. However, this tumour cell autonomous approach will be very challenging as gene delivery to every tumour cell will be very hard to achieve and unmodified cells will outgrow the modified ones. Moreover, many tumours are widely disseminated at the time of diagnosis, so gene delivery would need to be systemic.

Therefore, gene therapy approaches that create a systemic environment that is hostile to the tumour (e.g. by enhancing anti-tumour immunity) are more logical. Some forms of tumour immunity can be stimulated by infusion of monoclonal antibodies; for example, targeting receptors on tumour cells such as Her2 [73], or inhibiting feedback mechanisms in the immune system such as PD1/PD-L1 engagement [74,75]. However, there is clear evidence that links the number of effector T cells infiltrating tumours with clinical outcome [76]. So gene modification can be used to generate a large number of tumour-specific T cells, by engineering the patients’ own T cells to recognize the tumour. This has proven particularly effective in clinical trials when T cells are transduced with a lentiviral vector expressing a chimaeric antigen receptor that recognizes the haematopoietic surface protein CD19. The chimaeric receptor has a single chain antibody external domain and a series of T-cell receptor (TCR) signalling domains on the cytoplasmic tail (reviewed in [77]). The advantage of this approach is that, unlike the TCR itself, the chimaeric receptor works in all patients regardless of HLA genotype. However, it does depend on targeting T cells to a surface protein that is either tumour-specific or expressed only on normal cells that are not essential. Direct infusion of large numbers of activated, tumour-targeted T cells may well prove easier than trying to generate similar numbers of effector T cells by vaccination in tumour patients whose immune system is often suppressed. It should be noted that insertional oncogenesis has never been observed in gene therapy applications where T cells have been transduced by MLV or lentiviral vectors.

Initial proposals for gene therapy for AIDS involved strategies to inhibit virus replication, for example, by delivery of dominant negative viral proteins to HIV-infected cells [78]. Another idea was to couple an HIV-regulated promoter to a cytotoxic gene so that virally infected cells would be killed [79]. However, subsequent understanding of AIDS pathogenesis suggested that a better aim would be to supply cells that are resistant to HIV infection. This was elegantly demonstrated by bone marrow transplantation of an HIV-infected individual with marrow from an individual homozygous for a relatively common deletion in the HIV receptor CCR5, which resulted in an apparent HIV cure [80]. The first gene therapy clinical trial to use genome editing took T cells from HIV-infected individuals and used a ZFN to target the HIV-binding site on CCR5. In this initial study, one patient who was heterozygous for the CCR5 deletion had a reduced HIV level after therapy, which suggests that more efficient editing of both alleles will improve the effect [8]. Also, if a gene editing protocol were to be carried out on bone marrow stem cells, then off-target effects, which could potentially be oncogenic, would need to be minimized.

## 6. Gene therapy as a different formulation of a conventional medicine

As already discussed in the case of haemophilia B, gene delivery may be a more convenient, reliable and cost-effective method of supplying biological medicines that are required systemically. Indeed the first licensed gene therapy medicine alipogene tiparvovec (Glybera) is an AAV1 vector carrying human lipoprotein lipase (LPL), to be injected intramuscularly for the treatment of patients with LPL deficiency. There has recently been much interest in using antibody gene delivery to muscle for the treatment of infectious diseases. In the case of a persistent virus such as HIV, permanent expression of a broadly neutralizing antibody could provide more effective protection than vaccination, which has thus far failed. The efficacy of this has been demonstrated in a humanized mouse model [7]. This technology has also been tested in mice for influenza prophylaxis, delivered either to muscle [81] or intranasally [82]. Here there may also be an advantage over vaccination, particularly in the elderly who do not mount an effective response to influenza vaccination. Antibody gene therapy as prophylaxis might also be very effective in a rapidly spreading pandemic, where vaccination might be too slow, for example, to protect key health workers. In these types of application, a clinically compatible small molecule that could regulate gene expression *in vivo* would be very useful. The antibiotic selection systems used *in vitro* (such as the ‘Tet on’ system [83]) are unsuitable *in vivo* because the bacterial tetracycline-controlled transactivator is immunogenic, leading to transduced cell elimination. A modified human protein that responds to a clinically suitable small molecule would be a great advance in this field.

Gene delivery also permits generation of active drugs at the site where they are needed. For example, Parkinson's disease is caused by a deficiency in dopamine in the brain, so dopaminergic neurons die leading to loss of movement control and debilitating tremors. A common treatment for Parkinson's disease is a tablet delivering a dopamine precursor combined with a drug to enhance blood–brain barrier permeability. This works well in early-stage disease but declines in efficacy. A gene therapy approach, delivering the enzymes to synthesize dopamine directly to the brain, might provide more stable local dopamine concentration. The first clinical trial used a lentiviral vector and reported safety and some efficacy [84], but more efficient delivery or higher gene expression will be necessary for full evaluation.

There have been a number of attempts to improve tissue vascularization after cardiac ischaemia by delivery of genes encoding vascular growth factors; however, clinical results have been disappointing [85]. Recently, local gene delivery using AAV1 encoding a sarcoplasmic reticulum calcium ATPase (SERCA2A) gene has reported promising results in patients with heart failure [86]. An improvement in contractile function was reported as well as an unexpected reduction in arrhythmia. Further trials of both AAV1 and adenovirus delivery of SERCA2A are under way. In the case of lung disease, gene therapy has concentrated on gene replacement of CFTR in cystic fibrosis patients. A number of small trials demonstrated expression of CFTR and local restoration of chloride conductance. However, the only trial looking for clinical effect, using aerosolized AAV2, did not demonstrate improved lung function [87]. Gene delivery to the lung epithelium is particularly challenging in cystic fibrosis patients due to mucus deposits.

Gene therapy can also be used to improve the efficacy of existing drugs. For example, temozolomide (TMZ) is used to treat glioblastoma, usually in combination with O6-benzylguanine (O6BG) to inhibit methylguanine methyltransferase (MGMT). MGMT is over-expressed by many glioblastomas and inactivates TMZ. Unfortunately, the amount of O6BG that can be used is limited because it is very toxic to haematopoietic cells. Gene therapy has been used to express the O6BG-resistant MGMT mutant P140 K in haematopoietic cells of glioblastoma patients, which has allowed them to receive more intensive chemotherapy [88].

## 7. Where will gene therapy be in another 25 years?

While it is 350 years since the founding of the Royal Society, it is only 25 years since the feasibility of gene transfer to patients was first demonstrated [18]. So in the last 25 years, considerable progress has been made and a number of gene therapy applications have provided benefit to patients in clinical trials. If we look forward it seems likely that proteins that are required systemically for prolonged periods may well be delivered by gene therapy. Antibodies are prime candidates for this type of gene delivery, to prevent infectious disease or provide rapid prophylaxis, and also to treat cancer or autoimmune disease. In the latter cases, where the antibodies recognize human molecules, local gene delivery and production of antibody, for example, at sites of tumour metastases or in inflamed joints in rheumatoid arthritis, might reduce systemic side effects. Gene regulation by clinically compatible small molecules may also be used to fine-tune the dose. Other familiar biological medicines may be replaced by gene therapy; for example, if reliable glucose-regulated expression of an insulin gene can be achieved then gene therapy can also be used to treat type 1 diabetes.

Many inherited monogenic disorders that have been treated by bone marrow transplantation with virally modified cells may in future be amenable to gene correction, although this requires bespoke treatments to correct different mutations. Gene editing may also be used for the treatment of monogenic eye conditions. For the most common inherited monogenic disorders, such as cystic fibrosis or the muscular dystrophies, effective gene therapy is likely to remain a delivery challenge. That is because there is not yet a simple way to deliver genes to a significant proportion of cells in tissues such as the lung epithelium or skeletal muscle. Many commercial gene therapy activities are likely to focus in the long term on gene addition therapy for common diseases such as heart disease or cancer. Injectable vectors are also more attractive as licensed medicines, because they can be manufactured and distributed in the conventional manner. For treatments requiring *ex vivo* cell modification, however, there will be a continued requirement for local production of gene-modified cells. Better production and purification methods for viral vectors are required; many current protocols are based on scale-up of research laboratory methods.
